# SilkDB 3.0: visualizing and exploring multiple levels of data for silkworm

**DOI:** 10.1093/nar/gkz919

**Published:** 2019-10-23

**Authors:** Fang Lu, Zhaoyuan Wei, Yongjiang Luo, Hailong Guo, Guoqing Zhang, Qingyou Xia, Yi Wang

**Affiliations:** Biological Science Research Center, Southwest University, Chongqing 400715, China

## Abstract

SilkDB is an open-accessibility database and powerful platform that provides comprehensive information on the silkworm (*Bombyx mori*) genome. Since SilkDB 2.0 was released 10 years ago, vast quantities of data about multiple aspects of the silkworm have been generated, including genome, transcriptome, Hi-C and pangenome. To visualize data at these different biological levels, we present SilkDB 3.0 (https://silkdb.bioinfotoolkits.net), a visual analytic tool for exploring silkworm data through an interactive user interface. The database contains a high-quality chromosome-level assembly of the silkworm genome, and its coding sequences and gene sets are more accurate than those in the previous version. SilkDB 3.0 provides a view of the information for each gene at the levels of sequence, protein structure, gene family, orthology, synteny, genome organization and gives access to gene expression information, genetic variation and genome interaction map. A set of visualization tools are available to display the abundant information in the above datasets. With an improved interactive user interface for the integration of large data sets, the updated SilkDB 3.0 database will be a valuable resource for the silkworm and insect research community.

## INTRODUCTION

The silkworm, *Bombyx mori*, which was domesticated during the last ∼5000 years from a wild progenitor, *Bombyx mandarina*, is one of the most economically important insects and the foundation of sericulture. It has been widely used as a bioreactor to produce recombinant proteins and other biomaterials (1). The silkworm has many basic physiological processes typical of insects, which have been conserved through insect evolution, and a considerable number of genes that are homologous to those of humans; thus, it has been widely used in various life science studies (2). The silkworm is considered a central model species for lepidopteran genomics and genetics, and it is second only to the fruit fly (*Drosophila melanogaster*) (3) as an insect model for biological studies.

In 2004, both Chinese and Japanese teams completed separate draft silkworm genomes (4,5). Subsequently, SilkDB was published in the *Nucleic Acids Research* database issue in 2005 (6), and its updated version (SilkDB 2.0) was released in 2010 (7). This online resource has greatly facilitated functional genomics research in silkworm and other insects. Thousands of users from more than eighty countries have analyzed their data with this database, and studies have cited SilkDB more than 400 times (Google Scholar).

Due to the importance of sericulture in agriculture and the flourishing development of next generation sequencing, a variety of omics studies of the silkworm have been performed over the last decade and generated multiple levels of data. For example, whole silkworm genome resequencing was performed using long reads (8), transcriptome analyses of silkworm tissues have identified several genes of interest for silk fiber formation (9,10), and recently, researchers carried out the first proteogenomics study of the silkworm using large-scale mass spectrometry to improve silkworm genome annotation (11). To facilitate the utilization of the rapidly growing genomic and other omic data associated with the silkworm, several databases have been released, including KAIKObase (12), SilkBase (8), BmTEdb (13), BmncRNAdb (14) and SilkPathDB (15). Though these databases have added to the available resources for silkworm genomic research, no platform yet incorporates multiple levels of silkworm data and provides integrated search, analysis, and visualization features through a single portal.

To better encompass all these data and develop a more user-friendly visual interface, we updated SilkDB to version 3.0 (https://silkdb.bioinfotoolkits.net), which offers substantial performance improvements over the previous version, including increased data, an upgraded visual interface and more tools. In SilkDB 3.0, the quality of the assembly has been significantly improved. The genome sequence is assigned to 28 chromosomes with a length of ∼468.3 Mb and includes 16 069 protein-coding genes. Moreover, the database contains a large amount of transcriptome data for exploring silkworm gene expression in different tissues. To more comprehensively characterize the genetic variation in silkworms, SilkDB 3.0 provides pangenome data analysis of 163 samples from different locations, allowing comparison of single nucleotide polymorphisms (SNPs) and insertion-deletions (indels). In addition, the database supplies Hi-C (high-throughput chromatin conformation capture) data obtained from six silkworm tissue types to aid understanding of gene regulatory mechanisms. Based on the above data, SilkDB 3.0 is mainly divided into the following modules: Gene-Info, eFP, Cell, Coexpression, 3D, Gene Family, Pan, JBrowse, Chr, Exp-Cube, Synteny, Hi-C and Ortholog. These modules can clearly display genetic data at diverse levels, and they are connected with each other. SilkDB 3.0 not only encompasses the basic functions of a searchable genome sequence but also combines several data visualization tools into the same interface, so users can explore multiple levels of biological data and comprehensively analyze genes.

## GENOME DATA AND ANNOTATION

In SilkDB 3.0, we have updated the silkworm genome assembly, which consists of 28 chromosomes encompassing ∼468.3 Mb. To do that, we first downloaded PacBio long reads (NCBI SRA: DRX058175) for the silkworm genome with an estimated depth of coverage of over 140×. The PacBio data were error-corrected and assembled using Canu (V1.5) (16). Then, to construct chromosome-scale scaffolds, Hi-C data (NCBI SRA: SRP220287) were first analyzed by the Hic-Pro pipeline (V2.11.1) (17), and then, Juicer (V1.5.6) was used to categorize and order these assemblies (18). Compared with the previous version (43 622 scaffolds of ∼432 Mb) (7) and the silkworm genome in SilkBase (∼460.3 Mb) (8) using QUAST (V5.02) (19), the genome sequence in SilkDB 3.0 is a high-quality, chromosome-level assembly that is more intact (Supplementary Figure S1A). The dot plot shows that these genome sequences are very similar with good synteny (Supplementary Figure S1B, C). Based on the new high-quality genomic data and transcriptome data (NCBI SRA: SRP219634), we reannotated and obtained gff3 files using MAKER (V2.31.10) (20). A total of 16 069 protein-coding genes were annotated, which is far more than the previous 14 623 genes and similar to the number in a recent report (16 880 genes) (8). The protein sequences were annotated against KO (21), GO (22), KOG (23), Pfam (24) and KEGG ENZYME (21) databases using local BLAST (V2.8.1) program with an *E*-value threshold of 1e−10. The genes were grouped into different families based on their protein domains using the Pfam database (24). OrthoVenn2 (25) was used for protein clustering and ortholog identification.

Moreover, we gathered 253 RNA-seq datasets from different tissues and stages of silkworm, including the instar, larval, wandering, pupal and moth stages (NCBI SRA: SRP219634). The RNA-seq data were first trimmed with fastp (V0.19.5) (26) and then aligned to the silkworm genome with HISAT2 (V2.1.0) (27) with the default parameters. The alignment results were converted, sorted and stored in bam file format with SAMtools (V1.9) (28). We calculated the expression values of each gene with an R package (ballgown (V2.16.0)) (29) and their coexpression relationships using Pearson's correlation coefficient.

To perform pangenome analysis, we collected a total of 163 silkworm strains, including 40 genome datasets from our previous study (30) and 123 genome datasets from Xiang's paper (31). These silkworm strains include 130 domestic and 33 wild silkworms. The raw reads were cleaned with fastp (V0.19.5) (26) and then aligned to our updated reference genome using BWA-MEM (V0.7.10) (32) with the default parameters. SNP calling and indel detection were performed with BCFtools (V0.1.19) (28).

## MAIN INTERFACE AND SEARCH FUNCTION

SilkDB 3.0 is a web-based tool combining a MySQL database management system with a dynamic web interface which was written with Python, HTML, CSS, Javascript and jQuery. The entire project is open access for anyone to use and is configured on an Ubuntu (V18.04) Linux machine with an Apache2 server.

**Figure 1.**
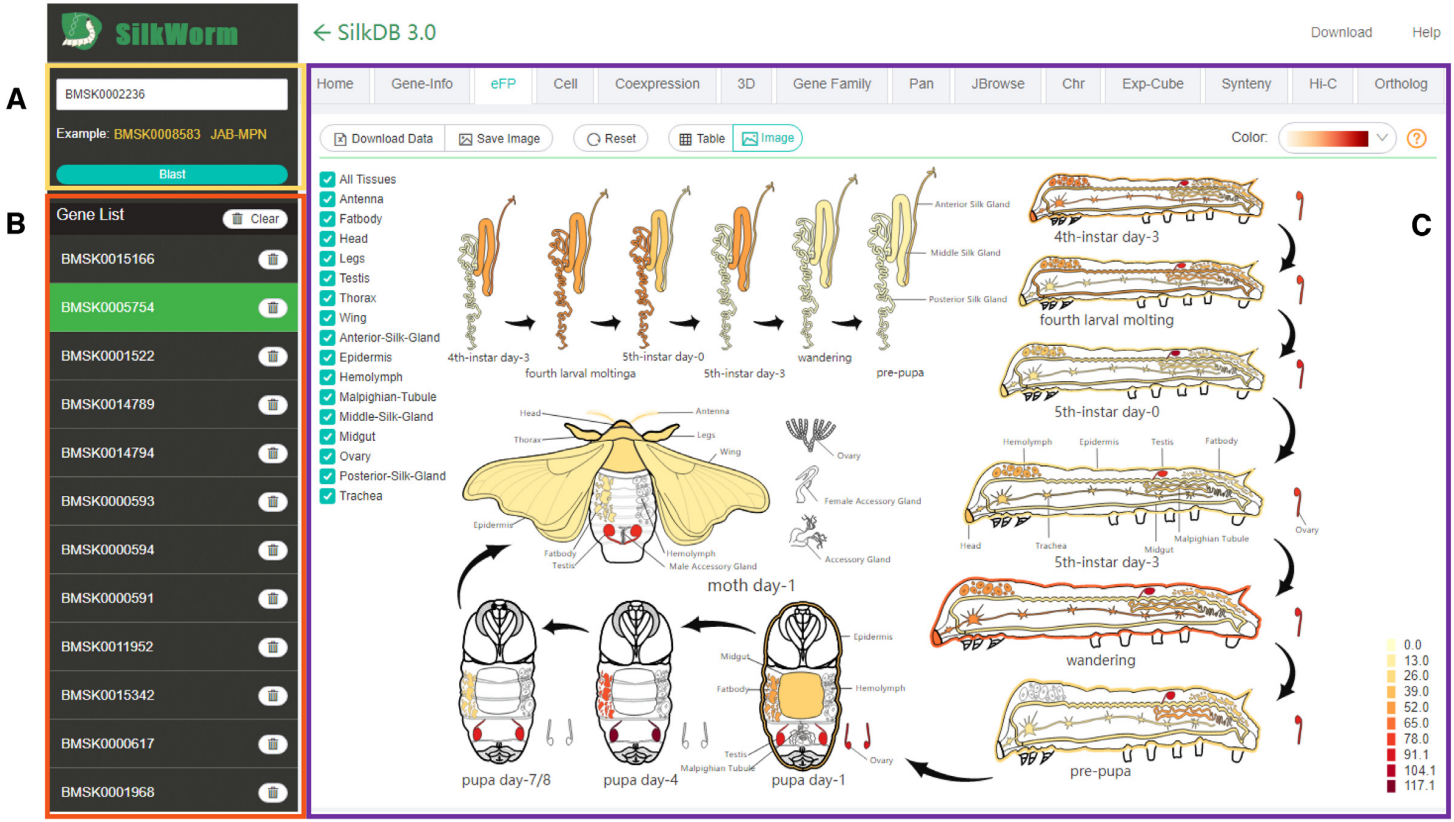
The main interface of SilkDB 3.0. (**A**) search panel, (**B**) gene panel, (**C**) module viewer panel.

The main interface for SilkDB 3.0 has three main elements: the search panel and the gene panel on the left and the module viewer panel on the right (Figure 1). Although SilkDB 3.0 contains many functional modules and a large quantity of information, its interface is simple and user-friendly. There are two ways to utilize the functional modules of the database to investigate genes. One way is to input keywords such as gene identifier (ID) or gene description to search for the gene of interest, after which the gene of interest will be shown in the gene panel. Another is to use the Blast function; the Blast result will show the genes in the database that are similar to the input sequence. Users can click the gene ID on the results page, and it will be added to the gene panel. Once the gene is displayed in the panel, a data loading management script sends queries to the database to retrieve information for each of the functional modules to display.

## GENE INFORMATION

**Figure 2.**
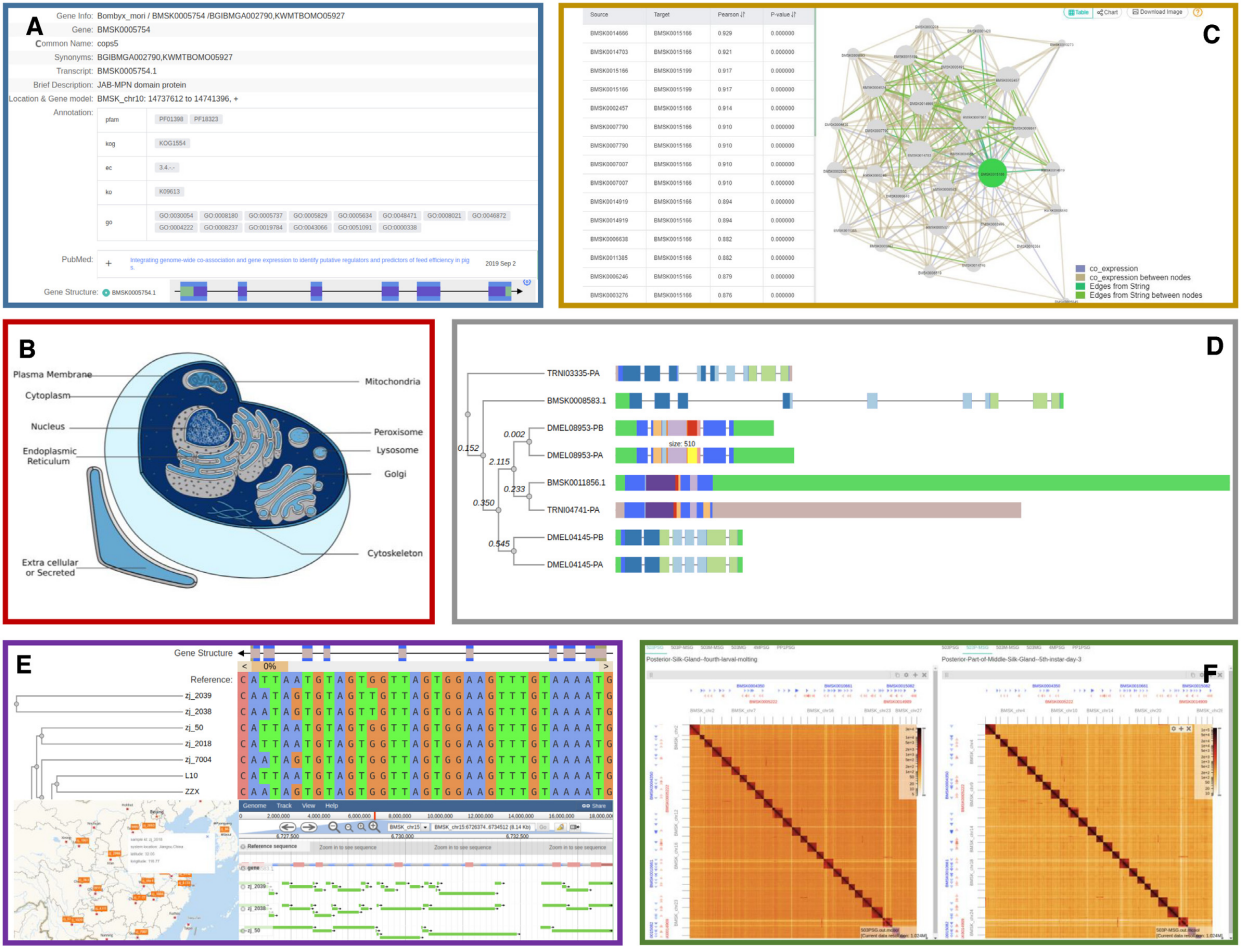
The main functional modules of SilkDB 3.0. (**A**) Gene-Info, (**B**) Cell eFP, (**C**) Coexpression, (**D**) Gene Family, (**E**) Pangenome, (**F**) Hi-C.

The Gene-Info module shows basic information about multiple aspects of the selected genes, such as gene ID, synonyms, protein domain description, gene distribution, and gene location. In addition, some functional annotations are drawn from multiple databases (Figure 2A). Moreover, the exons, untranslated regions (UTRs) and introns are shown to represent the gene architecture of the selected gene. In addition, the genomic, CDS and protein sequences are displayed on the page. Clicking an element in the gene structure viewer will highlight it in the sequence. Based on the sequence on the page, a primer design function is available, and users can select a sequence region to design primers for PCR experiments.

## eFP

The eFP (electronic fluorescent pictograph) viewer displays expression patterns by dynamically coloring the tissues of a pictographic representation according to gene expression levels (Figure 1C). In SilkDB 3.0, the eFP module presents the expression values of selected genes in 19 tissues during various growth stages (instar, larval, wandering, pupal, moth) with different colors and shades. Users can select which organs to display and save the picture in PNG format. In addition, users can choose the color system to change the display style for the eFP module.

In Cell eFP (Figure 2B), the database shows the predicted subcellular localization of proteins using ngLOC, an n-gram-based Bayesian classifier that predicts the subcellular localization of proteins in both prokaryotes and eukaryotes (33). The Cell eFP Viewer displays the predicted localization of a gene within a picture of a cell with a color gradient representing a confidence score that the selected gene will be found in a given compartment. This module helps researchers conduct genetic investigations at the subcellular level.

## COEXPRESSION

Gene coexpression networks are a powerful approach for detecting genes with similar expression patterns across large amounts of transcriptome data, clustering coexpressed genes that are most likely functionally related and speculating about the functions of uncharacterized genes (34). In SilkDB 3.0, we predicted gene coexpression relationships based on transcriptome data and generated a coexpression network. The Coexpression module provides a view of coexpressed genes by displaying them in a network with an interactive analysis tool (Figure 2C). The green node represents the selected gene, and the gray nodes indicate coexpressed genes. The node size indicates the number of adjacent nodes, allowing for easier discovery of network hub genes. The edge thickness of the connecting lines between the genes represents the weight value of the linked genes. The network also incorporates silkworm protein-protein association data from the STRING database (35). Users can click any gene in the network to add that gene to the gene panel. In addition, information about the coexpressed gene pairs that are displayed in the network is listed in a table.

## GENE FAMILY

Gene families often show some variation in terms of exon–intron structures, which provide valuable information for clarifying their evolutionary relationships. Thus, the structural information of genes and gene families can serve as material for phylogenetic analyses to understand gains, losses and changes in gene structure (36). To investigate the evolution of silkworm genes, SilkDB 3.0 contains a comprehensive gene comparison and evolution dataset with all the annotated genes in *Aedes aegypti*, *Drosophila melanogaster*, *Spodoptera litura*, *Tribolium castaneum*, *Trichoplusia ni* and *Bombyx mori*. The method for generating the trees and collecting the gene structure information are based on the PIECE database study (36,37). The gene family module provides a user-friendly graphical view that displays the gene structure and Pfam domain pattern diagram linked to a bootstrapped similarity dendrogram (Figure 2D). Users can collapse and expand the tree by clicking the nodes and clicking on the button in the upper right corner to modify the display style of the elements. Moreover, the diagram can be directly downloaded as a high-quality PNG format file.

## PANGENOME

Pangenomes, which capture a broad representation of the genomic variation contained in a gene pool (38,39), represent an especially useful resource for research and breeding (40). To more comprehensively characterize the genetic variation in the silkworm genome, we collected 163 different geographically representative samples from two published studies (30,31). By comparing them to the reference silkworm assembly, we uncovered a substantial number of SNPs and indels, which contain potentially important genetic information pertaining to silkworm evolution.

The Pangenome module consists of three parts: the multiple sequence alignment (MSA) viewer, the JBrowse (V1.16.6) (41) interface and a chart of the geographic location of each sample (Figure 2E). The viewer contains a phylogenetic tree browser at the left and an MSA at the right. Users can click the node ID in the tree to show the detailed information for the sample and analyze the variations in the selected gene in the viewer. The MSA contains SNPs between the reference genome and the samples, while indel data are displayed in the JBrowse interface. The JBrowse page also contains a tree displaying the relationship of the samples. Users can select multiple nodes, track their types (insertion, deletion, coverage or SNP) and then click the ‘Commit’ button to visualize these silkworm accessions in the genome browser. Moreover, the Pangenome module shows the geographic distribution of the samples on a world map.

## GENOME INTERACTION MAP

Three-dimensional (3D) chromatin structure plays an important role in gene regulatory mechanisms (42). Hi-C is a widely used conformation capture method that allows the capture of all-to-all chromatin contacts in a genome-wide manner. However, there is no online resource for silkworms that provides chromatin interaction partners for queried genes with genomic annotations. To overcome these limitations, we used HiGlass (43) to support multiscale contact maps and genomic data tracking visualization across multiple resolutions and loci for six silkworm Hi-C datasets (Figure 2F). The HiGlass viewer shows the genomic interactions with the selected gene as a heatmap. Users can smoothly and interactively browse the Hi-C heatmaps, zoom in and out to view different resolutions, and visualize maps showing the gene location. In addition, this viewer allows the synchronous comparison of two maps. In general, the HiGlass module will help researchers discover 3D genome organization and assist in deciphering the functions of silkworm gene regulation.

## OTHER TOOLS

**Figure 3.**
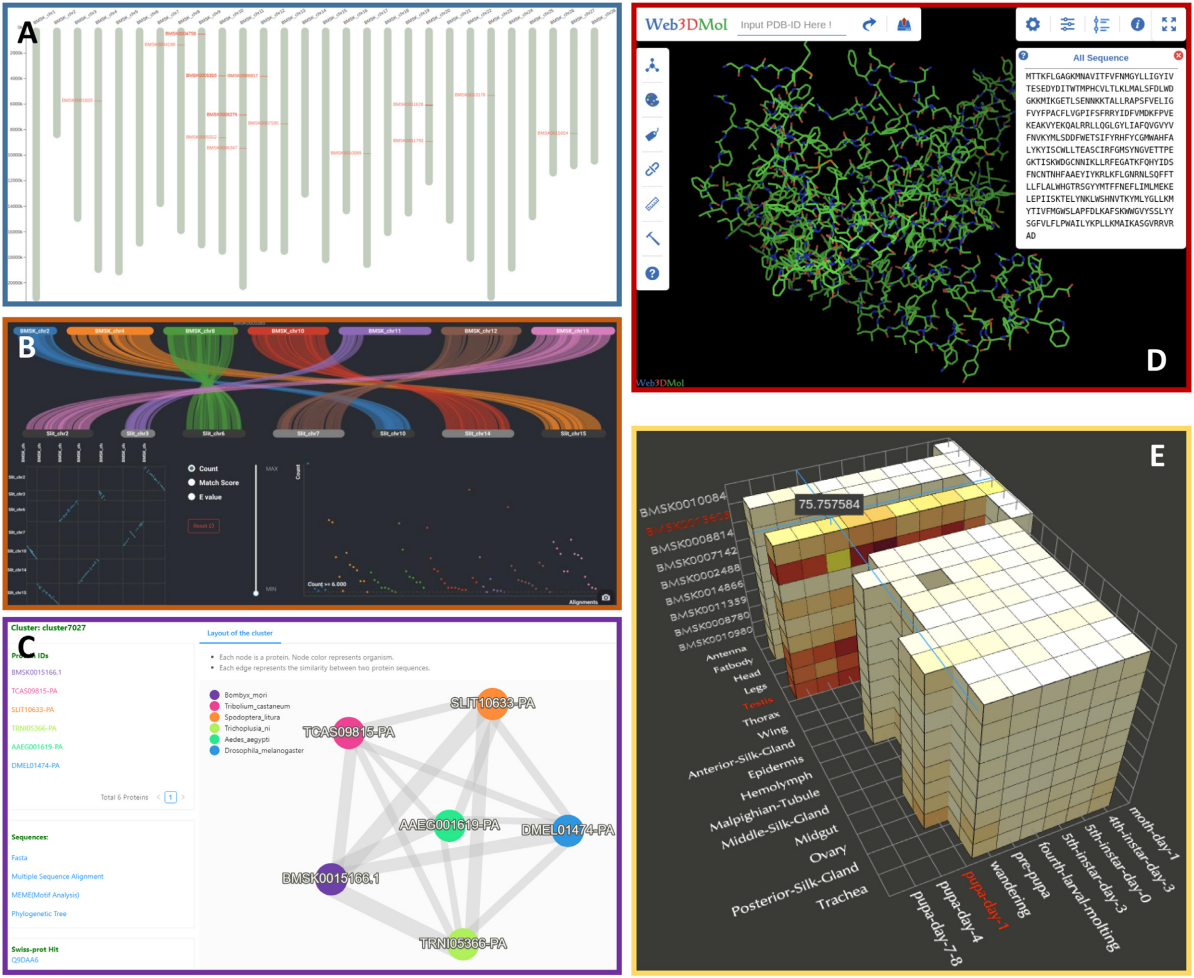
The other tools in SilkDB 3.0. (**A**) Chromosome Viewer, (**B**) Synteny Viewer, (**C**) Ortholog Cluster, (**D**) 3D structure, (**E**) Expression Cube.

SilkDB 3.0 also provides other powerful and user-friendly tools. The chromosome viewer (Figure 3A) supplies a simple way to display the site of the selected gene and its family on the 28 chromosomes. The Synteny module shows the collinearity of chromosomes between silkworms and other insect species (Figure 3B). The Ortholog module provides links about which ortholog clusters contain the selected gene (Figure 3C). The Molecule Viewer displays a 3D model of the protein molecular structure for the selected gene (Figure 3D). The protein structures are generated by Phyre2 (44). In addition, the Multigene expression module presents an ‘Expression Cube’ and heatmap to display the expression profile of the selected gene, together with the genes with the most highly correlated expression profiles (Figure 3E).

## DISCUSSION

At present, most insect genomes and genetic data are stored in multiple databases (45). Among them, SilkDB plays an important role in managing, sharing and mining biological data for silkworm. Using silkworm genome data, SilkDB provides significant insight into the biology and breeding of this species. Moreover, comprehensive studies of the silkworm genome using this database have reported fundamental information about its genome structure and the evolutionary rearrangement of chromosomes after domestication events (30). The growth of the insect community and the many biological processes being interrogated using silkworm as a model system (2) require the development of tools to access multiple levels of biological data. Integrating data from different biological levels can allow novel hypotheses to be generated (46). SilkDB 3.0 greatly improves the accuracy of silkworm genome assembly and annotation and integrates a large amount of silkworm data at multiple levels. The database allows users to analyze the different aspects of a gene in the silkworm genome and combine the results of the analyses. We hope that the capabilities of SilkDB 3.0 will provide researchers with many hypotheses for designing molecular biology experiments and will help to elucidate the functions of silkworm genes.

Furthermore, we will continue to improve the quality of the assembly and annotations of the silkworm genome sequence. At the same time, we plan to augment the available silkworm data to make the website information more comprehensive, including some new types of biological data, such as phenotype and metabonomics data. In addition to the frequently updated silkworm genome information, we hope to add other lepidopteran genomes to SilkDB and develop new comparative genomics visualization tools for these genomes.

## Supplementary Material

gkz919_Supplemental_File

## References

[1] Goldsmith M.R., Shimada T., Abe H. The genetics and genomics of the silkworm, Bombyx mori. Annu. Rev. Entomol. 2005; 50:71–100. 15355234 10.1146/annurev.ento.50.071803.130456

[2] Meng X., Zhu F., Chen K. Silkworm: a promising model organism in life science. J. Insect Sci. 2017; 17:97. 10.1093/jisesa/iex064 PMC5633739 29117372

[3] Adams M.D., Celniker S.E., Holt R.A., Evans C.A., Gocayne J.D., Amanatides P.G., Scherer S.E., Li P.W., Hoskins R.A., Galle R.F. et al. The genome sequence of Drosophila melanogaster. Science. 2000; 287:2185–2195. 10731132 10.1126/science.287.5461.2185

[4] Xia Q., Zhou Z., Lu C., Cheng D., Dai F., Li B., Zhao P., Zha X., Cheng T., Chai C. et al. A draft sequence for the genome of the domesticated silkworm (Bombyx mori). Science. 2004; 306:1937–1940. 15591204 10.1126/science.1102210

[5] Mita K., Kasahara M., Sasaki S., Nagayasu Y., Yamada T., Kanamori H., Namiki N., Kitagawa M., Yamashita H., Yasukochi Y. et al. The genome sequence of silkworm, Bombyx mori. DNA Res. 2004; 11:27–35. 15141943 10.1093/dnares/11.1.27

[6] Wang J., Xia Q., He X., Dai M., Ruan J., Chen J., Yu G., Yuan H., Hu Y., Li R. et al. SilkDB: a knowledgebase for silkworm biology and genomics. Nucleic Acids Res. 2005; 33:D399–D402. 15608225 10.1093/nar/gki116 PMC540070

[7] Duan J., Li R., Cheng D., Fan W., Zha X., Cheng T., Wu Y., Wang J., Mita K., Xiang Z. et al. SilkDB v2.0: a platform for silkworm (Bombyx mori) genome biology. Nucleic Acids Res. 2010; 38:D453–D456. 19793867 10.1093/nar/gkp801 PMC2808975

[8] Kawamoto M., Jouraku A., Toyoda A., Yokoi K., Minakuchi Y., Katsuma S., Fujiyama A., Kiuchi T., Yamamoto K., Shimada T. High-quality genome assembly of the silkworm, Bombyx mori. Insect Biochem. Mol. Biol. 2019; 107:53–62. 30802494 10.1016/j.ibmb.2019.02.002

[9] Hu W., Chen Y., Lin Y., Xia Q. Developmental and transcriptomic features characterize defects of silk gland growth and silk production in silkworm naked pupa mutant. Insect Biochem. Mol. Biol. 2019; 111:103175. 31150761 10.1016/j.ibmb.2019.05.010

[10] Shi R., Ma S.Y., He T., Peng J., Zhang T., Chen X.X., Wang X.G., Chang J.S., Xia Q.Y., Zhao P. Deep insight into the transcriptome of the single silk gland of Bombyx mori. Int. J. Mol. Sci. 2019; 20:2491. 10.3390/ijms20102491 PMC6567255 31137550

[11] Ye X., Tang X., Wang X., Che J., Wu M., Liang J., Ye L., Qian Q., Li J., You Z. et al. Improving silkworm genome annotation using a proteogenomics approach. J. Proteome Res. 2019; 18:3009–3019. 31250652 10.1021/acs.jproteome.8b00965

[12] Shimomura M., Minami H., Suetsugu Y., Ohyanagi H., Satoh C., Antonio B., Nagamura Y., Kadono-Okuda K., Kajiwara H., Sezutsu H. et al. KAIKObase: an integrated silkworm genome database and data mining tool. BMC Genomics. 2009; 10:486. 19843344 10.1186/1471-2164-10-486 PMC2770533

[13] Xu H.E., Zhang H.H., Xia T., Han M.J., Shen Y.H., Zhang Z. BmTEdb: a collective database of transposable elements in the silkworm genome. Database. 2013; 2013:bat055. 23886610 10.1093/database/bat055 PMC3722987

[14] Zhou Q.Z., Zhang B., Yu Q.Y., Zhang Z. BmncRNAdb: a comprehensive database of non-coding RNAs in the silkworm, Bombyx mori. BMC Bioinformatics. 2016; 17:370. 27623959 10.1186/s12859-016-1251-y PMC5022206

[15] Li T., Pan G.Q., Vossbrinck C.R., Xu J.S., Li C.F., Chen J., Long M.X., Yang M., Xu X.F., Xu C. et al. SilkPathDB: a comprehensive resource for the study of silkworm pathogens. Database. 2017; 2017:bax001. 10.1093/database/bax001 PMC5467577 28365723

[16] Koren S., Walenz B.P., Berlin K., Miller J.R., Bergman N.H., Phillippy A.M. Canu: scalable and accurate long-read assembly via adaptive k-mer weighting and repeat separation. Genome Res. 2017; 27:722–736. 28298431 10.1101/gr.215087.116 PMC5411767

[17] Servant N., Varoquaux N., Lajoie B.R., Viara E., Chen C.J., Vert J.P., Heard E., Dekker J., Barillot E. HiC-Pro: an optimized and flexible pipeline for Hi-C data processing. Genome Biol. 2015; 16:259. 26619908 10.1186/s13059-015-0831-x PMC4665391

[18] Durand N.C., Shamim M.S., Machol I., Rao S.S., Huntley M.H., Lander E.S., Aiden E.L. Juicer provides a one-click system for analyzing loop-resolution Hi-C experiments. Cell Syst. 2016; 3:95–98. 27467249 10.1016/j.cels.2016.07.002 PMC5846465

[19] Gurevich A., Saveliev V., Vyahhi N., Tesler G. QUAST: quality assessment tool for genome assemblies. Bioinformatics. 2013; 29:1072–1075. 23422339 10.1093/bioinformatics/btt086 PMC3624806

[20] Cantarel B.L., Korf I., Robb S.M., Parra G., Ross E., Moore B., Holt C., Sanchez Alvarado A., Yandell M. MAKER: an easy-to-use annotation pipeline designed for emerging model organism genomes. Genome Res. 2008; 18:188–196. 18025269 10.1101/gr.6743907 PMC2134774

[21] Kanehisa M., Furumichi M., Tanabe M., Sato Y., Morishima K. KEGG: new perspectives on genomes, pathways, diseases and drugs. Nucleic Acids Res. 2017; 45:D353–D361. 27899662 10.1093/nar/gkw1092 PMC5210567

[22] The Gene Ontology, C. The gene ontology resource: 20 years and still GOing strong. Nucleic Acids Res. 2019; 47:D330–D338. 30395331 10.1093/nar/gky1055 PMC6323945

[23] Koonin E.V., Fedorova N.D., Jackson J.D., Jacobs A.R., Krylov D.M., Makarova K.S., Mazumder R., Mekhedov S.L., Nikolskaya A.N., Rao B.S. et al. A comprehensive evolutionary classification of proteins encoded in complete eukaryotic genomes. Genome Biol. 2004; 5:R7. 14759257 10.1186/gb-2004-5-2-r7 PMC395751

[24] El-Gebali S., Mistry J., Bateman A., Eddy S.R., Luciani A., Potter S.C., Qureshi M., Richardson L.J., Salazar G.A., Smart A. et al. The Pfam protein families database in 2019. Nucleic Acids Res. 2019; 47:D427–D432. 30357350 10.1093/nar/gky995 PMC6324024

[25] Xu L., Dong Z., Fang L., Luo Y., Wei Z., Guo H., Zhang G., Gu Y.Q., Coleman-Derr D., Xia Q. et al. OrthoVenn2: a web server for whole-genome comparison and annotation of orthologous clusters across multiple species. Nucleic Acids Res. 2019; 47:W52–W58. 31053848 10.1093/nar/gkz333 PMC6602458

[26] Chen S., Zhou Y., Chen Y., Gu J. fastp: an ultra-fast all-in-one FASTQ preprocessor. Bioinformatics. 2018; 34:i884–i890. 30423086 10.1093/bioinformatics/bty560 PMC6129281

[27] Kim D., Langmead B., Salzberg S.L. HISAT: a fast spliced aligner with low memory requirements. Nat. Methods. 2015; 12:357–360. 25751142 10.1038/nmeth.3317 PMC4655817

[28] Li H., Handsaker B., Wysoker A., Fennell T., Ruan J., Homer N., Marth G., Abecasis G., Durbin R. Genome Project Data Processing, S. The Sequence Alignment/Map format and SAMtools. Bioinformatics. 2009; 25:2078–2079. 19505943 10.1093/bioinformatics/btp352 PMC2723002

[29] Frazee A.C., Pertea G., Jaffe A.E., Langmead B., Salzberg S.L., Leek J.T. Ballgown bridges the gap between transcriptome assembly and expression analysis. Nat. Biotechnol. 2015; 33:243–246. 25748911 10.1038/nbt.3172 PMC4792117

[30] Xia Q., Guo Y., Zhang Z., Li D., Xuan Z., Li Z., Dai F., Li Y., Cheng D., Li R. et al. Complete resequencing of 40 genomes reveals domestication events and genes in silkworm (Bombyx). Science. 2009; 326:433–436. 19713493 10.1126/science.1176620 PMC3951477

[31] Xiang H., Liu X., Li M., Zhu Y., Wang L., Cui Y., Liu L., Fang G., Qian H., Xu A. et al. The evolutionary road from wild moth to domestic silkworm. Nat. Ecol. Evol. 2018; 2:1268–1279. 29967484 10.1038/s41559-018-0593-4

[32] Li H., Durbin R. Fast and accurate short read alignment with Burrows-Wheeler transform. Bioinformatics. 2009; 25:1754–1760. 19451168 10.1093/bioinformatics/btp324 PMC2705234

[33] King B.R., Vural S., Pandey S., Barteau A., Guda C. ngLOC: software and web server for predicting protein subcellular localization in prokaryotes and eukaryotes. BMC Res Notes. 2012; 5:351. 22780965 10.1186/1756-0500-5-351 PMC3532370

[34] Stuart J.M., Segal E., Koller D., Kim S.K. A gene-coexpression network for global discovery of conserved genetic modules. Science. 2003; 302:249–255. 12934013 10.1126/science.1087447

[35] Szklarczyk D., Morris J.H., Cook H., Kuhn M., Wyder S., Simonovic M., Santos A., Doncheva N.T., Roth A., Bork P. et al. The STRING database in 2017: quality-controlled protein-protein association networks, made broadly accessible. Nucleic Acids Res. 2017; 45:D362–D368. 27924014 10.1093/nar/gkw937 PMC5210637

[36] Wang Y., You F.M., Lazo G.R., Luo M.C., Thilmony R., Gordon S., Kianian S.F., Gu Y.Q. PIECE: a database for plant gene structure comparison and evolution. Nucleic Acids Res. 2013; 41:D1159–D1166. 23180792 10.1093/nar/gks1109 PMC3531150

[37] Wang Y., Xu L., Thilmony R., You F.M., Gu Y.Q., Coleman-Derr D. PIECE 2.0: an update for the plant gene structure comparison and evolution database. Nucleic Acids Res. 2017; 45:1015–1020. 10.1093/nar/gkw935 PMC5210635 27742820

[38] Tettelin H., Masignani V., Cieslewicz M.J., Donati C., Medini D., Ward N.L., Angiuoli S.V., Crabtree J., Jones A.L., Durkin A.S. et al. Genome analysis of multiple pathogenic isolates of Streptococcus agalactiae: implications for the microbial “pan-genome”. Proc. Natl. Acad. Sci. U.S.A. 2005; 102:13950–13955. 16172379 10.1073/pnas.0506758102 PMC1216834

[39] Medini D., Serruto D., Parkhill J., Relman D.A., Donati C., Moxon R., Falkow S., Rappuoli R. Microbiology in the post-genomic era. Nat. Rev. Microbiol. 2008; 6:419–430. 18475305 10.1038/nrmicro1901

[40] Hubner S., Bercovich N., Todesco M., Mandel J.R., Odenheimer J., Ziegler E., Lee J.S., Baute G.J., Owens G.L., Grassa C.J. et al. Sunflower pan-genome analysis shows that hybridization altered gene content and disease resistance. Nat. Plants. 2019; 5:54–62. 30598532 10.1038/s41477-018-0329-0

[41] Buels R., Yao E., Diesh C.M., Hayes R.D., Munoz-Torres M., Helt G., Goodstein D.M., Elsik C.G., Lewis S.E., Stein L. et al. JBrowse: a dynamic web platform for genome visualization and analysis. Genome Biol. 2016; 17:66. 27072794 10.1186/s13059-016-0924-1 PMC4830012

[42] Dekker J., Marti-Renom M.A., Mirny L.A. Exploring the three-dimensional organization of genomes: interpreting chromatin interaction data. Nat. Rev. Genet. 2013; 14:390–403. 23657480 10.1038/nrg3454 PMC3874835

[43] Kerpedjiev P., Abdennur N., Lekschas F., McCallum C., Dinkla K., Strobelt H., Luber J.M., Ouellette S.B., Azhir A., Kumar N. et al. HiGlass: web-based visual exploration and analysis of genome interaction maps. Genome Biol. 2018; 19:125. 30143029 10.1186/s13059-018-1486-1 PMC6109259

[44] Kelley L.A., Mezulis S., Yates C.M., Wass M.N., Sternberg M.J. The Phyre2 web portal for protein modeling, prediction and analysis. Nat. Protoc. 2015; 10:845–858. 25950237 10.1038/nprot.2015.053 PMC5298202

[45] Li F., Zhao X., Li M., He K., Huang C., Zhou Y., Li Z., Walters J.R. Insect genomes: progress and challenges. Insect Mol. Biol. 2019; doi:10.1111/imb.12599. 10.1111/imb.12599 31120160

[46] Waese J., Fan J., Pasha A., Yu H., Fucile G., Shi R., Cumming M., Kelley L.A., Sternberg M.J., Krishnakumar V. et al. ePlant: visualizing and exploring multiple levels of data for hypothesis generation in plant biology. Plant Cell. 2017; 29:1806–1821. 28808136 10.1105/tpc.17.00073 PMC5590499

