# Supplementary material for: SilkDB 3.0: visualizing and exploring multiple levels of data for silkworm
Source: Nucleic Acids Res. 2019 Oct 23;48(D1):D749–55. doi: 10.1093/nar/gkz919 (PMC7145608; doi:10.1093/nar/gkz919)
Supplement: gkz919_Supplemental_File [file gkz919_supplemental_file.pdf]

Worst Median Best

Show heatmap

Statistics without reference	version2	version3	silkbase
# contigs	40 255	28	28
# contigs (>= 0 bp)	43 462	28	28
# contigs (>= 1000 bp)	4782	28	28
# contigs (>= 5000 bp)	1278	28	28
# contigs (>= 10000 bp)	808	28	28
# contigs (>= 25000 bp)	449	28	28
# contigs (>= 50000 bp)	298	28	28
Largest contig	16 203 812	21 484 951	21 465 692
Total length	480 482 162	454 710 009	445 114 022
Total length (>= 0 bp)	481 803 763	454 710 009	445 114 022
Total length (>= 1000 bp)	456 852 213	454 710 009	445 114 022
Total length (>= 5000 bp)	450 223 819	454 710 009	445 114 022
Total length (>= 10000 bp)	446 886 634	454 710 009	445 114 022
Total length (>= 25000 bp)	441 208 018	454 710 009	445 114 022
Total length (>= 50000 bp)	435 845 159	454 710 009	445 114 022
N50	4 008 358	17 604 390	16 840 672
N75	1 585 222	15 122 178	14 801 489
L50	38	12	12
L75	87	19	19
GC (%)	37.66	38.32	38.33
**Mismatches**			
# N's	50 095 828	42 400	452 530
# N's per 100 kbp	10 426	9.32	101.67

Supplement Fig. 1A Compared with the previous version (43,622 scaffolds of ~432Mb) and a silkworm genome in Silkbase (~460.3Mb) using QUAST(V5.02), the genome sequence in SilkDB 3.0 is a high-quality, chromosome-level assembly and more intact.

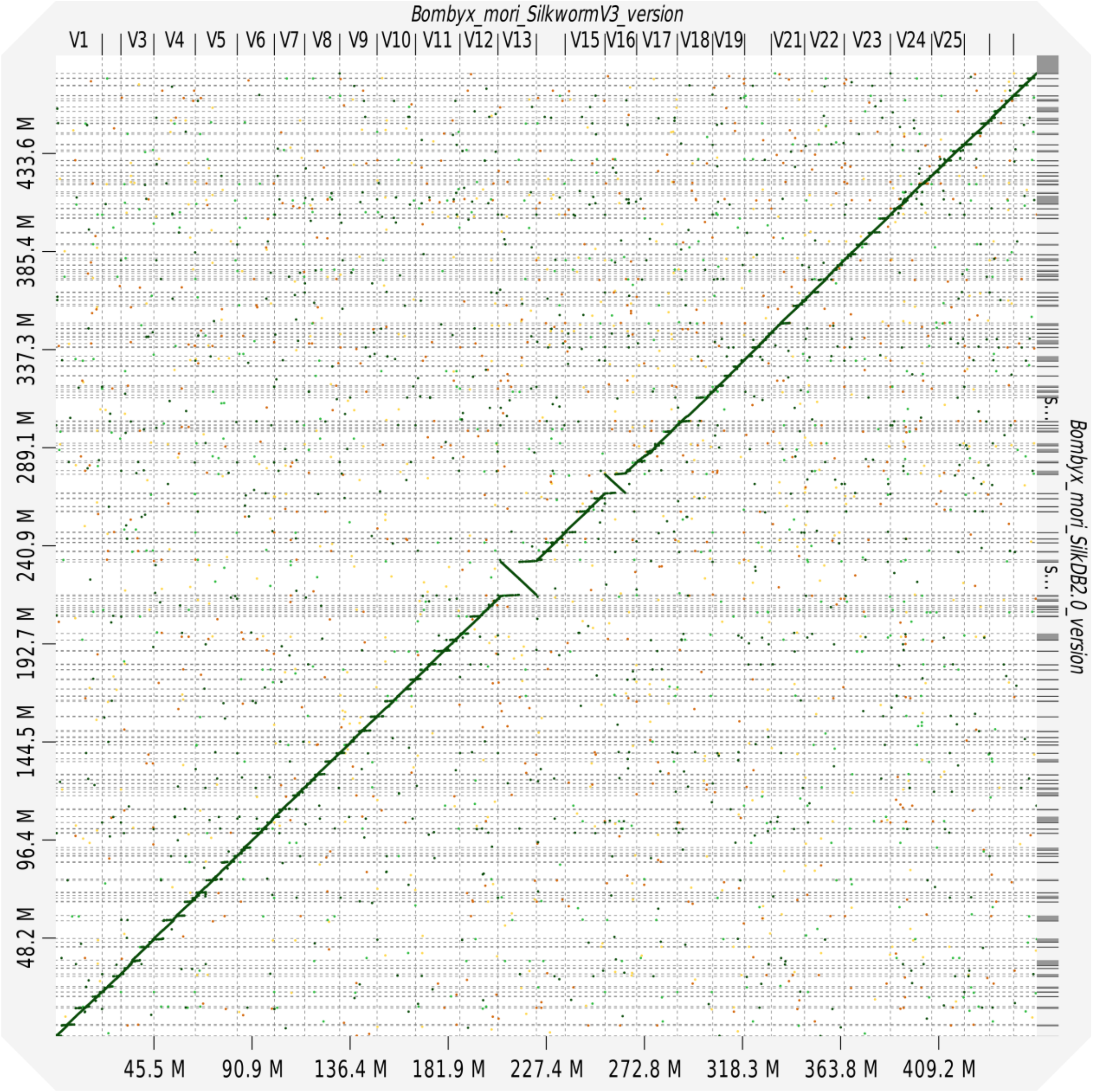

Supplement Fig. 1B. The dotplot comparison between genome sequences (Silkdb 3.0 versus Silkdb 2.0).

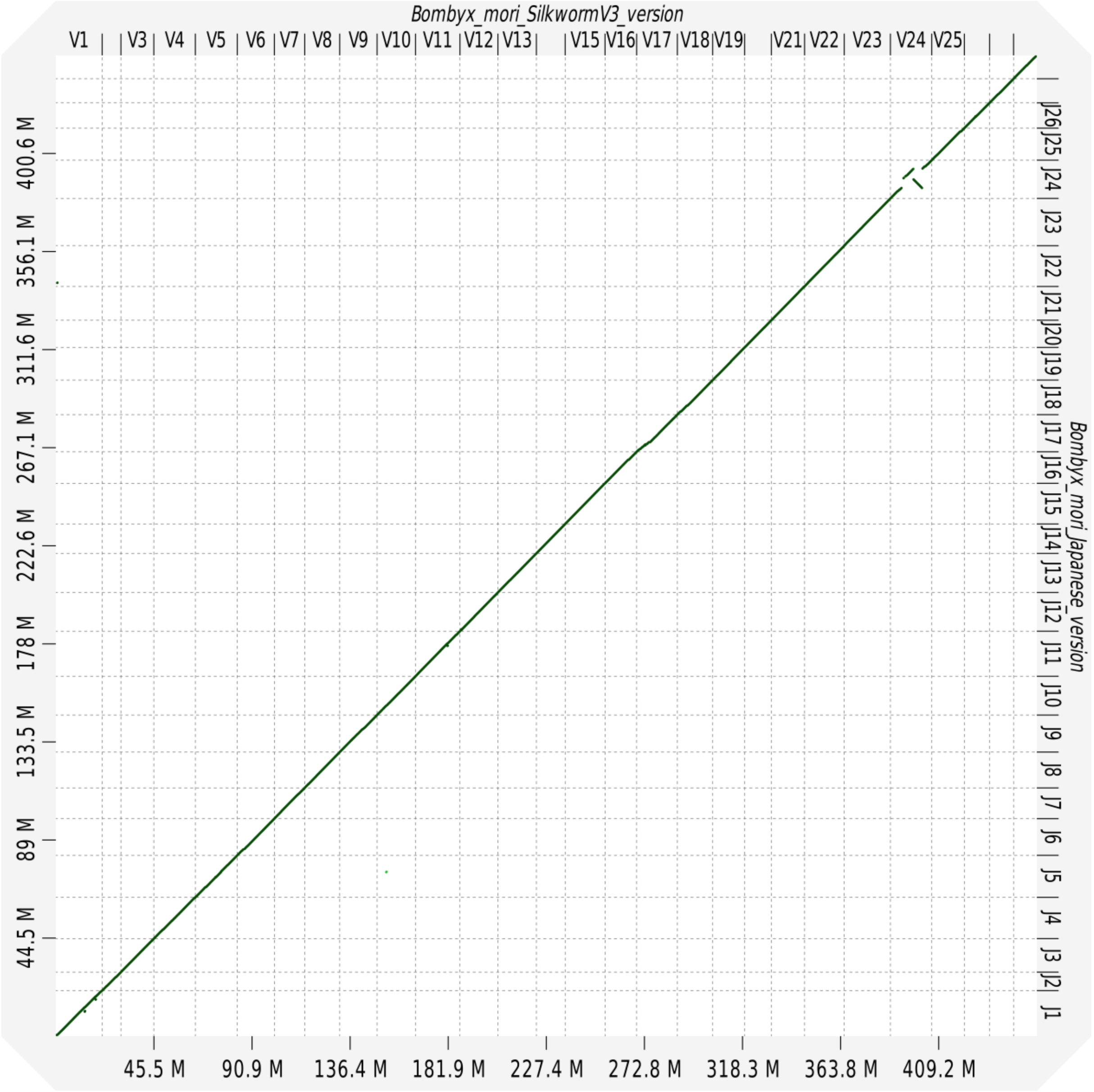

Supplement Fig. 1C. The dotplot comparison between genome sequences (Silkdb 3.0 versus SilkBase).
